# Platelet and Doppler ultrasound signatures of histological fibrosis in chronic liver disease: a correlation study based on Ishak staging

**DOI:** 10.1007/s40477-026-01126-y

**Published:** 2026-02-22

**Authors:** Andrea Boccatonda, Maria Chiara Garlisi, Alice Brighenti, Marco Musmeci, Livia Masi, Sofia Maria Bakken, Carla Serra

**Affiliations:** Diagnostic and Therapeutic Interventional Ultrasound Unit, IRCCS Azienda Ospedaliero-Universitaria di Bologna, Policlinico Sant’Orsola-Malpighi, Via Massarenti N 9, 40138 Bologna, Italy

**Keywords:** Hepatic fibrosis, Doppler ultrasound, Platelet count, Ishak score, Portal hypertension, Splenic index

## Abstract

**Background and aims:**

Non-invasive assessment of hepatic fibrosis remains a major challenge in clinical hepatology. Platelet count and Doppler ultrasound parameters have been proposed as indirect markers of portal hypertension and fibrotic remodeling, yet their relationship with histological fibrosis severity across the full disease spectrum is not fully defined. This study aimed to evaluate the associations between platelet count (PLT) and Doppler ultrasound parameters along the histological continuum of hepatic fibrosis assessed by the Ishak staging system.

**Methods:**

In this retrospective study, 120 patients with biopsy-proven hepatic fibrosis were analyzed. Doppler ultrasound examinations were performed just before the liver biopsy. Assessed parameters included portal vein caliber, mean portal vein velocity, hepatic arterial resistive and pulsatility indices, splenic arterial indices, and splenic area. PLT was obtained from routine blood tests. Associations between PLT, Doppler parameters, and Ishak fibrosis stage were evaluated using Spearman’s rank correlation. Subgroup analyses were conducted across Ishak stages 0–2 (mild fibrosis), 3–4 (moderate fibrosis), and 5–6 (advanced fibrosis/cirrhosis).

**Results:**

Platelet count showed a strong inverse correlation with Ishak fibrosis stage (*ρ* = – 0.55, *q* < 0.01). Significant inverse associations were also observed between platelet count and key Doppler markers of portal hypertension, including portal vein caliber (*ρ* = – 0.46, *q* < 0.05) and splenic area (*ρ* = – 0.51, *q* < 0.05). Hepatic and splenic arterial resistive and pulsatility indices demonstrated weaker positive correlations with fibrosis severity (*ρ* ≈ + 0.30 to 0.40), which did not consistently reach statistical significance after correction for multiple testing. Correlations between platelet count and portal vein caliber as well as splenic area were significantly stronger in patients with advanced fibrosis compared with those with mild fibrosis (*p*_diff < 0.05).

**Conclusions:**

PLT and selected Doppler ultrasound parameters change in parallel with histological fibrosis severity, reflecting the progressive development of portal hypertension and vascular remodeling. These findings support a combined, trend-based interpretation of PLT and Doppler ultrasound markers as accessible, non-invasive indicators of advanced liver disease, rather than as stand-alone diagnostic tools.

## Introduction

Chronic liver disease (CLD) represents a major global health burden, affecting more than 100 million individuals worldwide and accounting for a substantial proportion of liver-related morbidity and mortality. The progressive accumulation of hepatic fibrosis, ultimately leading to cirrhosis and portal hypertension, is the key determinant of prognosis, therapeutic decision-making, and surveillance strategies in these patients [1, 2]. Accurate assessment of fibrosis severity is therefore crucial for both clinical management and risk stratification. Liver biopsy remains the reference standard for fibrosis staging; however, its invasive nature, risk of complications, sampling variability, and limited suitability for longitudinal monitoring have driven the search for reliable non-invasive alternatives [3]. Over the past two decades, serum biomarkers and elastographic techniques have improved fibrosis assessment, yet their diagnostic accuracy decreases in intermediate stages of disease and may be influenced by inflammatory activity, cholestasis, congestion, or obesity [4, 5]. Consequently, easily accessible and widely reproducible non-invasive markers continue to be needed, particularly in routine clinical practice and in resource-limited settings. Doppler ultrasound offers a dynamic and non-invasive evaluation of hepatic and splanchnic hemodynamics, reflecting both intrahepatic vascular resistance and extrahepatic adaptations to portal hypertension. Progressive fibrosis is associated with increased sinusoidal resistance, distortion of the vascular architecture, and compensatory arterialization of hepatic blood flow [6]. These pathophysiological changes translate into measurable Doppler parameters, including enlargement of portal vein caliber, reduction of portal flow velocity, and increased resistive and pulsatility indices of the hepatic and splenic arteries [7]. Several studies have demonstrated that Doppler-derived indices correlate with portal pressure and clinical severity of cirrhosis, supporting their role as indirect markers of advanced liver disease [7, 8]. In parallel, thrombocytopenia is a well-established hematological hallmark of chronic liver disease. Its pathogenesis is multifactorial, involving splenic sequestration due to portal hypertension, reduced hepatic thrombopoietin synthesis, and bone marrow suppression in advanced disease [9]. Platelet count has consistently shown an inverse relationship with fibrosis stage and portal hypertension and has been incorporated into several non-invasive fibrosis scores [10, 11]. However, platelet count alone lacks sufficient specificity to fully characterize the hemodynamic consequences of fibrotic progression.

Histological staging systems remain essential for validating non-invasive markers. The Ishak scoring system, with its expanded 0–6 scale, allows a more granular assessment of fibrosis progression compared with simpler classifications and is particularly suitable for correlation analyses across the full spectrum of disease severity [12]. Despite this, relatively few studies have systematically examined the relationship between platelet count, Doppler ultrasound parameters, and histologically defined fibrosis stages using the Ishak system.

The present study was therefore designed to investigate the association between platelet count and Doppler ultrasound parameters across the entire histological spectrum of hepatic fibrosis, using Ishak staging as the reference standard. By integrating hematological and hemodynamic markers, we aimed to evaluate their combined potential as practical, non-invasive indicators of fibrosis severity and portal hypertension in patients with chronic liver disease.

## Materials and methods

### Study design and population

This was a retrospective, observational study conducted at Diagnostic and Therapeutic Interventional Ultrasound Unit, Bologna University Hospital (Italy). We reviewed clinical, laboratory, histological, and Doppler ultrasound data from patients with chronic liver disease who underwent percutaneous liver biopsy between January 2018 and September 2025.

Inclusion criteria were:

- (i) age ≥ 18 years;
- (ii) biopsy-proven chronic liver disease with available Ishak fibrosis staging;
- (iii) Color-Doppler ultrasound examination performed within 3 months of liver biopsy;
- (iv) availability of complete platelet count data.

Exclusion criteria included:

- (i) active gastrointestinal bleeding at the time of evaluation;
- (ii) hematological disorders affecting platelet count unrelated to portal hypertension;
- (iii) presence of hepatocellular carcinoma or other hepatic malignancies;
- (iv) portal vein thrombosis;
- (v) previous splenectomy;
- (vi) incomplete or non-standardized Doppler ultrasound measurements.

### Histological assessment

Percutaneous liver biopsies were obtained under ultrasound guidance using a 18-gauge needle. Biopsy specimens were considered adequate if ≥ 15 mm in length and containing at least 6 portal tracts. All samples were independently evaluated by experienced hepatopathologists blinded to clinical and Doppler data. Fibrosis was staged according to the Ishak scoring system, ranging from stage 0 (no fibrosis) to stage 6 (established cirrhosis).

For subgroup analyses, patients were stratified as follows:

- Ishak 0–2: mild fibrosis.
- Ishak 3–4: moderate fibrosis.
- Ishak 5–6: advanced fibrosis/cirrhosis.

### Color-Doppler ultrasound examination

All patients underwent standardized color-Doppler ultrasound examination using a GE LOGIQ E10 ultrasound system (GE Healthcare), equipped with a 3.5-MHz convex transducer. Examinations were performed after an overnight fast, with patients in the supine or slight left lateral decubitus position. All Doppler evaluations were performed by a single experienced sonographer (> 5 years of expertise in hepatologic Doppler ultrasound), who was blinded to histological and laboratory data, in order to minimize inter-operator variability. Doppler measurements were obtained during quiet respiration, avoiding deep inspiration or Valsalva maneuvers. For each parameter, three consecutive measurements were acquired, and the mean value was used for the final analysis. The following parameters were assessed according to standardized methodology:

- Portal vein caliber: maximum internal diameter (mm), measured at the hepatic hilum.
- Portal vein mean velocity: time-averaged mean of the maximal flow velocity measured over the cardiac cycle (cm/s).
- Hepatic artery indices: resistive index (RI) and pulsatility index (PI) measured in the right and left hepatic artery branches.
- Splenic artery indices: resistive index (RISA) and pulsatility index (PISA).
- Splenic area: calculated in cm^2^ obtained on a standard splenic section.

### Laboratory data

Platelet count (PLT, × 10^9^/L) was obtained from complete blood counts performed on the same day as the Doppler ultrasound examination. Standard liver biochemical tests (AST, ALT, total bilirubin, albumin, INR) were collected for descriptive purposes but were not included in the primary correlation analyses.

### Statistical analysis

Continuous variables were tested for normality using the Shapiro–Wilk test and are presented as mean ± standard deviation or median (interquartile range), as appropriate. Given the non-normal distribution of most variables, non-parametric statistical methods were applied. Correlations between platelet count and Doppler ultrasound parameters were evaluated using Spearman’s rank correlation coefficient (*ρ*). Correlation analyses were performed both across the entire cohort and within predefined Ishak subgroups (0–2, 3–4, 5–6). Continuous trends between Ishak fibrosis stage (treated as an ordinal variable from 0 to 6) and Doppler or laboratory parameters were also assessed using Spearman correlation. Differences in correlation strength between Ishak subgroups were compared using Fisher’s *z*-transformation. To account for multiple comparisons, false discovery rate correction was applied using the Benjamini–Hochberg procedure, and adjusted *q* values are reported. All statistical analyses were performed using SPSS Statistics for Windows (IBM SPSS Statistics). A two-sided *p* value ≤ 0.05 was considered statistically significant.

Given the relatively limited sample size within Ishak subgroups—particularly in the advanced fibrosis group—and the strong pathophysiological interdependence among Doppler ultrasound variables reflecting portal hypertension, multivariable regression analyses were not performed. We considered that inclusion of multiple correlated Doppler parameters in a multivariable model would increase the risk of overfitting and unstable estimates, potentially limiting interpretability. Therefore, the analysis was intentionally focused on non-parametric, trend-based correlations anchored to histological staging.

### Ethical considerations

The study was conducted in accordance with the Declaration of Helsinki and approved by the local Institutional Review Board (CE AVEC: 676/2025/Tess/AOUBo). Given the retrospective nature of the study, the requirement for written informed consent was waived.

## Results

### Study population

A total of 120 patients with biopsy-proven chronic liver disease were included in the analysis. The study cohort comprised 65 men (54%) and 55 women (46%), with a mean age of 56 ± 12 years. The main etiologies of liver disease were viral hepatitis (*n* = 54, 45%), metabolic dysfunction-associated steatotic liver disease (MASLD) (*n* = 42, 35%), and autoimmune or cholestatic disorders (*n* = 24, 20%) (Table 1). According to histological staging, 40 patients (33%) had mild fibrosis (Ishak 0–2), 45 (37%) had moderate fibrosis (Ishak 3–4), and 35 (29%) had advanced fibrosis or cirrhosis (Ishak 5–6).

**Table 1:** Baseline demographic, laboratory, histological, and Doppler ultrasound characteristics of the biopsied cohort

Variable	All patients	Viral hepatitis	MASLD	Autoimmune/cholestatic
*N*	120	54	42	24
Age (years)	52.0 [42.0–61.0]	45.5 [41.3–50.5]	64.0 [64.0–72.0]	51.0 [39.3–56.5]
BMI (kg/m^2^)	27.4 [23.9–31.2]	23.7 [21.5–24.7]	31.5 [27.6–32.7]	21.4 [19.5–22.6]
Platelet count (× 10^9^/L)	220.0 [163.5–269.5]	239.0 [206.5–268.5]	231.0 [216.0–321.5]	254.0 [208.5–281.5]
AST (U/L)	96.0 [53.8–152.8]	261.0 [152.8–398.5]	74.0 [50.5–77.0]	103.0 [53.8–119.3]
ALT (U/L)	87.0 [47.0–194.8]	310.0 [194.8–770.3]	41.0 [39.5–61.5]	69.0 [47.0–118.0]
GGT (U/L)	97.0 [53.8–110.0]	103.0 [53.8–119.3]	105.0 [66.0–110.0]	85.0 [49.0–107.0]
ALP (U/L)	112.0 [98.0–140.0]	129.0 [104.8–221.5]	98.0 [83.5–98.0]	140.0 [111.0–210.0]
Bilirubin (mg/dL)	0.90 [0.60–1.36]	1.36 [0.75–1.68]	0.62 [0.43–0.67]	0.90 [0.60–1.40]
Albumin (g/L)	34.8 [27.4–36.0]	34.8 [4.9–35.9]	19.7 [12.1–27.4]	36.0 [33.0–40.0]
INR	1.01 [0.98–1.05]	1.02 [1.01–1.08]	0.96 [0.93–0.98]	1.01 [0.98–1.05]
Splenic area (cm^2^)	43.0 [33.1–58.0]	33.0 [32.9–35.0]	45.0 [37.3–50.8]	35.0 [32.2–40.7]
Hepatic artery RI (left)	0.69 [0.64–0.73]	0.70 [0.70–0.71]	0.69 [0.63–0.73]	0.69 [0.64–0.72]
Hepatic artery PI (right)	1.25 [1.06–1.48]	1.31 [1.17–1.41]	1.47 [1.47–1.47]	1.10 [0.96–1.25]
Splenic artery RI	0.59 [0.54–0.63]	0.54 [0.50–0.59]	0.60 [0.53–0.64]	0.57 [0.54–0.61]
Splenic artery PI	0.88 [0.77–1.00]	0.79 [0.68–0.93]	1.31 [1.31–1.31]	0.85 [0.79–0.93]

Data are reported as median [interquartile range]

### Platelet count and fibrosis stage

**Fig. 1:**
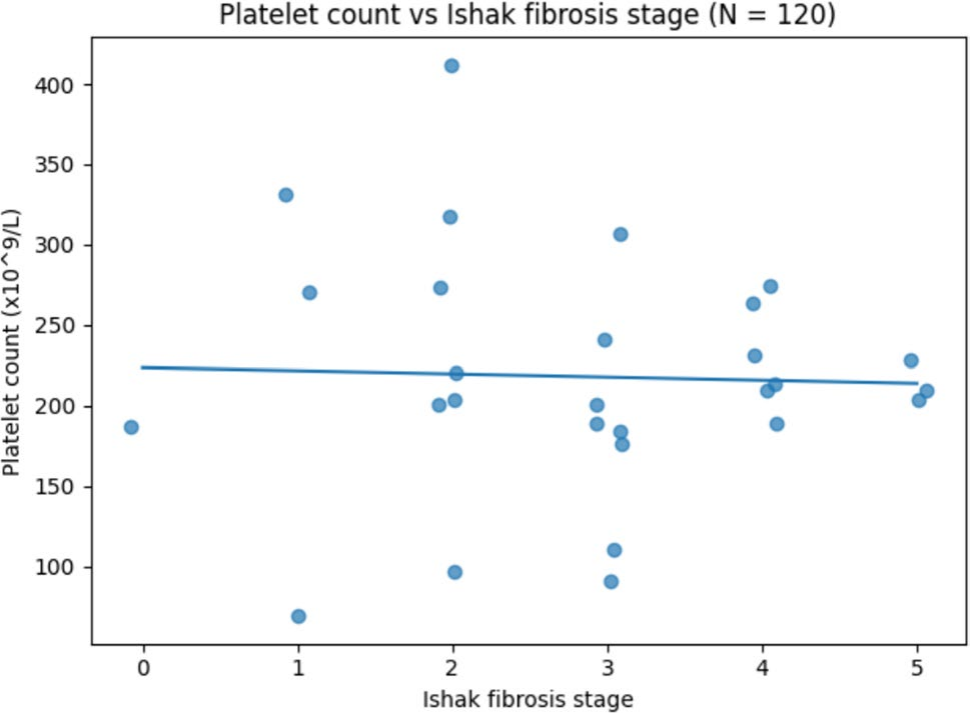
Scatter plot showing the relationship between platelet count and Ishak fibrosis stage in the study cohort (*N* = 120). Each point represents an individual patient. The solid line indicates the fitted trend, illustrating an overall inverse association between platelet count and increasing fibrosis severity

Platelet count showed a significant inverse correlation with fibrosis severity across the entire cohort. When Ishak stage was treated as an ordinal variable (0–6), platelet count progressively decreased with increasing fibrosis stage (*ρ* = – 0.55, *p* < 0.001, *q *< 0.01) (Fig. 1). Mean platelet values declined from 205 ± 45 × 10^9^/L in patients with mild fibrosis to 118 ± 40 × 10^9^/L in those with advanced fibrosis/cirrhosis.

### Correlation between platelet count and Doppler ultrasound parameters

Spearman correlation analyses between platelet count and Doppler ultrasound parameters were performed in the overall cohort and stratified by Ishak fibrosis stage (Table 2). In patients with mild fibrosis (Ishak 0–2), no significant correlations were observed between platelet count and Doppler parameters. In the moderate fibrosis group (Ishak 3–4), inverse correlations began to emerge between platelet count and portal vein caliber as well as splenic area, although these associations did not consistently reach statistical significance after false discovery rate (FDR) correction. In patients with advanced fibrosis or cirrhosis (Ishak 5–6), platelet count demonstrated significant inverse correlations with key Doppler indicators of portal hypertension. Specifically, platelet count correlated negatively with portal vein caliber (*ρ* = – 0.46, *q* < 0.05) and splenic area (*ρ* = – 0.51, *q* < 0.05). Weaker inverse associations were observed between platelet count and hepatic and splenic arterial indices (*ρ* ≈ – 0.30), which did not reach statistical significance after FDR correction. Comparison of correlation coefficients across fibrosis stages using Fisher’s *z*-transformation revealed a significant strengthening of the association between platelet count and both portal vein caliber and splenic area from mild to advanced fibrosis (*p*_diff < 0.05). Correlation coefficients and adjusted significance levels for the overall cohort are summarized in Table 2.

**Table 2:** Correlation between platelet count and Doppler ultrasound parameters

Parameter	*ρ* (PLT vs.)	*q*	Interpretation
Portal vein caliber	– 0.46	< 0.05	Larger portal diameter associated with thrombocytopenia
Splenic area	– 0.51	< 0.05	Splenomegaly linked to platelet sequestration
RIHA SN	– 0.32	< 0.1	Higher arterial resistance with lower PLT
PISA	– 0.29	NS	Weak negative trend

Spearman’s rank correlation coefficients (*ρ*) and false discovery rate–adjusted *q* values are reported

*PLT* platelet count, *RIHA* hepatic artery resistive index, *PISA* splenic artery pulsatility index

### Doppler ultrasound parameters and Ishak fibrosis stage

When Doppler ultrasound variables were directly correlated with Ishak fibrosis stage (0–6), several parameters showed significant trends across the spectrum of disease severity. Portal vein caliber increased progressively with advancing fibrosis (*ρ* = + 0.45, *p* = 0.003), while splenic area also demonstrated a strong positive correlation with Ishak stage (*ρ* = + 0.50, *p* = 0.001). Hepatic arterial resistive and pulsatility indices showed moderate positive correlations with fibrosis severity (*ρ* ≈ + 0.34 to 0.36), indicating increasing vascular resistance with advancing disease, although these associations were borderline after correction for multiple testing (Table 3). No significant correlation was observed between portal vein mean velocity and Ishak stage.

**Table 3:** Correlation between Ishak fibrosis stage and laboratory and Doppler ultrasound parameters

Variable	*ρ* (Ishak vs.)	*p*	*q*	Direction
Platelet count	– 0.55	< 0.001	< 0.01	↓ with fibrosis
Portal vein caliber	+ 0.45	0.003	0.04	↑ with fibrosis
Splenic area	+ 0.50	0.001	0.03	↑ with fibrosis
RIHA SN	+ 0.36	0.02	0.08	↑ with fibrosis
PIHADX	+ 0.34	0.03	0.09	↑ with fibrosis
Vel (portal velocity)	– 0.12	NS	–	Tendency to decrease

Spearman’s rank correlation coefficients (*ρ*), *p* values, and false discovery rate–adjusted *q* values are reported. Positive *ρ* values indicate increasing parameter values with advancing fibrosis stage, while negative *ρ* values indicate decreasing values

*RIHA* hepatic artery resistive index, *PIHADX* hepatic artery pulsatility index, *Vel* portal vein mean velocity

### Etiology-based subgroup analysis

An exploratory subgroup analysis suggested that the relationship between platelet count and Doppler ultrasound markers of portal hypertension may vary according to the underlying etiology of chronic liver disease. In patients with viral hepatitis, platelet count showed a strong inverse correlation with splenic area (*ρ* = – 0.67, *p* = 0.003), supporting a close link between thrombocytopenia and hypersplenism in this setting. In contrast, the association between platelet count and portal vein caliber was weaker and did not reach statistical significance (*ρ* = – 0.36, *p* = 0.18).

In patients with MASLD/MASH, no significant associations were observed between platelet count and Doppler parameters, including splenic area (*ρ* = + 0.32, *p* = 0.27) and portal vein caliber (*ρ* = + 0.27, *p* = 0.40). Similarly, in autoimmune and cholestatic liver diseases, platelet count did not correlate with Ishak fibrosis stage (*ρ* = + 0.21, *p* = 0.69) or splenic area (*ρ* = + 0.11, *p* = 0.66), although an inverse trend was observed with portal vein caliber (*ρ* = – 0.65, *p* = 0.08). Taken together, these findings suggest that the hematologic–hemodynamic interplay linking platelet count to portal hypertension-related Doppler changes may differ across etiological contexts. Given the limited sample size within subgroups, these results should be regarded as hypothesis-generating.

## Discussion

This study demonstrates that platelet count and Doppler ultrasound parameters show coherent, physiologically consistent associations with histological fibrosis severity, as graded by the Ishak score. The observed hematologic and hemodynamic changes reflect the progressive development of portal hypertension and vascular remodeling characteristic of chronic liver disease. Notably, these associations were strongest for Doppler markers directly related to portal hypertension, supporting their role as indirect indicators of advanced fibrotic disease. Thrombocytopenia is a well-recognized feature of chronic liver disease and arises from multiple mechanisms, including splenic sequestration secondary to portal hypertension, hypersplenism, and reduced hepatic thrombopoietin synthesis [13, 14]. In our cohort, platelet count showed a strong inverse correlation with Ishak fibrosis stage, confirming previous observations that declining platelet levels parallel fibrotic progression and increasing portal pressure. Notably, the progressive decline in platelet count across Ishak stages supports the concept that thrombocytopenia reflects the cumulative hemodynamic burden of chronic liver disease rather than isolated histological features. Similar associations have been reported in large clinical cohorts and have formed the basis for platelet-based non-invasive indices of fibrosis [11, 15, 16].

Doppler ultrasound provides dynamic information on the vascular consequences of fibrosis and portal hypertension. In this study, portal vein caliber and splenic area demonstrated the strongest positive correlations with Ishak stage, indicating progressive portal venous congestion and splenic enlargement with advancing fibrosis. These parameters represent structural manifestations of increased portal pressure and venous pooling and are less susceptible to short-term hemodynamic variability than flow velocity measurements. Hepatic arterial resistive and pulsatility indices showed moderate positive correlations with fibrosis severity, although these associations did not consistently reach statistical significance after correction for multiple testing. This finding aligns with the known pathophysiology of hepatic arterialization in advanced fibrosis, whereby increased intrahepatic resistance leads to compensatory changes in arterial flow. However, arterial indices are influenced by multiple systemic and technical factors, which may partly explain their weaker and less consistent associations compared with portal and splenic parameters. Portal vein mean velocity showed only a weak, non-significant tendency to decrease with advancing fibrosis, supporting previous observations that velocity measurements alone are relatively insensitive markers of fibrosis severity.

When platelet count was directly correlated with Doppler ultrasound parameters, significant inverse associations were observed with portal vein caliber and splenic area. These findings underscore the close link between thrombocytopenia and structural manifestations of portal hypertension. These associations should be interpreted as parallel pathophysiological trends rather than as independent effects, given the shared mechanistic background of portal hypertension. In contrast, weaker associations were observed with hepatic and splenic arterial indices, suggesting that platelet count more closely reflects venous congestion and splenic sequestration than arterial hemodynamic adaptations. Taken together, these results indicate that platelet count and Doppler markers of portal hypertension change in parallel along the continuum of fibrotic progression, reflecting shared underlying pathophysiological mechanisms.

Our findings are consistent with previous studies demonstrating the association between platelet count, spleen size, and portal hypertension. Giannini et al. showed that platelet count and splenic dimensions are strongly linked to clinically significant portal hypertension [10], while Kim et al. reported that Doppler-derived portal and splenic parameters correlate with disease severity in cirrhosis [7]. Similarly, Piscaglia et al. highlighted the value of Doppler ultrasound in capturing vascular remodeling associated with chronic liver disease, particularly for parameters reflecting portal venous congestion rather than arterial flow alone [6]. Compared with prior work, the present study adds value by anchoring Doppler and hematologic findings to histological staging using the Ishak score across the full spectrum of fibrosis, thereby strengthening the biological plausibility of these non-invasive associations. The etiology-specific patterns observed in this exploratory analysis highlight that thrombocytopenia may not uniformly reflect portal hypertension across all chronic liver diseases, underscoring the importance of considering etiological context when interpreting non-invasive markers.

### Limitations

This study has several limitations. Its retrospective, single-center design may limit generalizability, and external validation in independent cohorts is lacking. Doppler ultrasound measurements are operator-dependent, and although standardized acquisition protocols were used, transient hemodynamic fluctuations cannot be entirely excluded. In addition, subgroup analyses were exploratory and limited by sample size. Moreover, the absence of multivariable analyses represents a limitation of the present study; however, this choice was driven by the limited subgroup sample sizes and by the high collinearity among Doppler parameters, which could have produced unreliable or overfitted models. Future prospective, multicenter studies integrating Doppler ultrasound with elastography and clinical outcomes are warranted to further refine the role of these non-invasive markers.

## Conclusions

To the best of our knowledge, this is one of the few studies to systematically correlate platelet count and Doppler ultrasound parameters with histological fibrosis severity across the full Ishak staging spectrum, rather than dichotomizing fibrosis into cirrhosis versus non-cirrhosis. Platelet count and Doppler ultrasound parameters show robust and physiologically consistent correlations with Ishak fibrosis stage. Thrombocytopenia, portal vein dilation, and splenic enlargement represent accessible indicators of advanced fibrosis and portal hypertension. Integrating these measures could refine non-invasive fibrosis assessment, particularly where elastography or biopsy are unavailable.
